# The Effect of an Atherogenic Diet and Acute Hyperglycaemia on Endothelial Function in Rabbits Is Artery Specific

**DOI:** 10.3390/nu12072108

**Published:** 2020-07-16

**Authors:** Alexander Tacey, Tawar Qaradakhi, Cassandra Smith, Chris Pittappillil, Alan Hayes, Anthony Zulli, Itamar Levinger

**Affiliations:** 1 Institute for Health and Sport (iHeS), Victoria University, Melbourne, VIC 3011, Australia; alexander.tacey@live.vu.edu.au (A.T.); tawar.qaradakhi@live.vu.edu.au (T.Q.); cassandra.smith3@live.vu.edu.au (C.S.); chris.pittapillil@live.vu.edu.au (C.P.); alan.hayes@vu.edu.au (A.H.); anthony.zulli@vu.edu.au (A.Z.); 2 Australian Institute for Musculoskeletal Science (AIMSS), Department of Medicine-Western Health, Melbourne Medical School, The University of Melbourne, Melbourne, VIC 3021, Australia

**Keywords:** atherosclerosis, nitric oxide, nitrative stress, diabetes, immunohistochemistry

## Abstract

Hyperglycaemia has a toxic effect on blood vessels and promotes coronary artery disease. It is unclear whether the dysfunction caused by hyperglycaemia is blood vessel specific and whether the dysfunction is exacerbated following an atherogenic diet. Abdominal aorta, iliac, and mesenteric arteries were dissected from New Zealand White rabbits following either a 4-week normal or atherogenic diet (*n* = 6–12 per group). The arteries were incubated ex vivo in control or high glucose solution (20 mM or 40 mM) for 2 h. Isometric tension myography was used to determine endothelial-dependent vasodilation. The atherogenic diet reduced relaxation as measured by area under the curve (AUC) by 25% (*p* < 0.05), 17% (*p* = 0.06) and 40% (*p* = 0.07) in the aorta, iliac, and mesenteric arteries, respectively. In the aorta from the atherogenic diet fed rabbits, the 20 mM glucose altered EC_50_ (*p* < 0.05). Incubation of the iliac artery from atherogenic diet fed rabbits in 40 mM glucose altered EC_50_ (*p* < 0.05). No dysfunction occurred in the mesentery with high glucose incubation following either the normal or atherogenic diet. High glucose induced endothelial dysfunction appears to be blood vessel specific and the aorta may be the optimal artery to study potential therapeutic treatments of hyperglycaemia induced endothelial dysfunction.

## 1. Introduction

Type 2 diabetes is a major risk factor for cardiovascular complications, including atherosclerosis and subsequently coronary artery disease (CAD) [1,2]. Whilst diabetes and CAD can occur independently, diabetes often accelerates atherosclerosis development, increasing the risk of adverse cardiovascular events such as myocardial infarction [1]. The devastating effect of diabetes on the vascular system is caused, in part, by hyperglycaemia, which is characterised by toxic levels of circulating blood glucose [3,4].

Endothelial dysfunction is the first detectable sign of atherogenesis [5] and is a significant predictor of future cardiovascular events [6]. The impairment of nitric oxide (NO) mediated endothelial dependant vasodilation is a hallmark and one of the earliest indications of endothelial dysfunction [5]. Hyperglycaemia promotes endothelial dysfunction via a number of pathways, each of which are associated with a common link, the generation of reactive oxygen species (ROS), and oxidative/nitrative stress [7]. Specifically, hyperglycaemia induced mitochondrial electron transport system overproduction of superoxide binds with NO to produce peroxynitrite, reducing the bioavailability of NO and promoting endothelial dysfunction [8,9].

Acute elevations in circulating blood glucose, such as that which occurs in the post-prandial state, are a major risk factor for diabetes-induced endothelial dysfunction [10,11], perhaps more so than fasting blood glucose and haemoglobin A1c (HbA1c) [12]. A number of studies have reported that acute (2 to 6 h) ex vivo high glucose incubations can reduce endothelial-dependent vasodilation in arteries of rabbits [13,14,15] and rats [16,17,18,19]. However, no previous studies have completed high glucose incubations following a diet that mimics an atherosclerotic milieu, which is important to understand the effects of acute hyperglycaemia in a disease state. Furthermore, a study from our laboratory has shown that different vascular beds (thoracic aorta, renal, carotid, and iliac arteries) respond differently to hormonal stimulus, indicating that vascular beds are not homogeneous in their responses [20].

As such, the aim of this study was to determine if acute ex vivo high glucose incubations would impair endothelial function in aorta, iliac, and mesenteric arteries and whether the impairment would be exacerbated by an atherogenic diet. We hypothesised that high glucose incubations would reduce endothelium-dependant relaxation and that the impairment would be aggravated following an atherogenic diet.

## 2. Materials and Methods

### 2.1. Ethical Approval

This study was approved by the Victoria University Animal Ethics Committee (#14/005) and complied with the Australian National Health and Medical Research Council code for the care and use of animals for scientific purposes (8th edition).

### 2.2. Animal Model

Male New Zealand White rabbits (*n* = 6–12) at 3 months of age were randomly allocated into two groups and were fed a normal chow diet (Specialty Feeds, Glen Forrest, WA, Australia) or an atherogenic diet (a normal diet combined with 1% methionine, 0.5% cholesterol, and 5% peanut oil; SF00-218, Specialty Feeds, Glen Forrest, WA, Australia) for 4 weeks [21]. The animals were housed in separate cages on a 12 h light/dark cycle at a constant temperature of 21 °C. Food and water were supplied *ad libidum*.

### 2.3. Isometric Tension Myography

Following the 4-week diet, the rabbits were sedated with medetomidine (0.25 mL/kg), anaesthetised with 4% isoflurane, and exsanguinated via severing the inferior vena cava. The arterial system was immediately flushed with ice cold Krebs ((mM) 118 NaCl; 4.7 KCl; 1.2 MgSO_4_·7H_2_O; 1.2 KH_2_PO_4_; 25 NaHCO_3_; 1.25 CaCl and 11.7 glucose). The abdominal aorta (2 to 3 cm below the diaphragm), external iliac artery (immediately after the aortic bifurcation), and main mesenteric artery were excised, cleaned of connective tissue and fat, and cut into 3 mm rings. Blood vessel reactivity was measured via an isometric tension organ bath system (Zultek Engineering, Melbourne, Australia), as previously described [22,23]. Briefly, each vessel was incubated in physiological Krebs solution warmed to 37 °C and bubbled with 95% oxygen and 5% carbon dioxide. Following 30 min acclimatisation, the rings were strung up between 2 metal hooks attached to a force transducer to measure the tension of the vessel. Each vessel was passively stretched to a tension comparative to its size—the abdominal aorta to 2 g, the iliac artery to 1 g, and the mesenteric artery to 0.5 g. After 30 min, the vessels were again stretched to their respective tension for a further 30 min. Subsequently, the vessels were incubated in Krebs (11 mM glucose) or high glucose Krebs (20 mM or 40 mM glucose). Vasodilation of blood vessels in 11 mM glucose has previously been shown to cause relaxation equivalent to incubation in 5 mM glucose [14]. The respective Krebs solutions were refreshed every 30 min and incubated for a total of 2 h. Following the incubation, blood vessels were pre-contracted with 3 × 10^−7^ M phenylephrine (aorta and iliac artery) or 3 × 10^−7^ M cirazoline (mesenteric artery). Once the contraction reached a plateau, endothelium-dependant vasodilation was determined via a cumulative dose response curve to acetylcholine (ACh) in half-log increments (10^−8^ M to 10^−5^ M). Maximal relaxation (E_max_) was determined as the maximal dilation below the phenylephrine/cirazoline plateau. The log dose of ACh that produced half the maximal relaxation was reported as EC_50_. The area under the curve (AUC) was determined as the total area of relaxation below the phenylephrine/cirazoline plateau. Endothelial dysfunction was considered when there was an alteration to one or a combination of E_max_, EC_50_, and AUC that represented a reduction in the vasodilation of the blood vessels. All chemicals and reagents were supplied by Sigma Aldrich, St. Louis, MO, USA unless otherwise specified.

### 2.4. Immunohistochemistry (IHC)

The blood vessel rings were placed into 4% paraformaldehyde, left overnight, and then transferred into 1× phosphate buffered saline (PBS) at 4 °C. This was followed by paraffin processing (Microm STP120, Thermo Scientific, Waldorff, Germany) and embedding in paraffin blocks. Sections were cut at 5 μm, deparaffinised in xylene, rehydrated, and blocked with 1% goat serum in 10 mm TrisCl (pH 7.4) for 20 min. Primary mouse monoclonal anti-bodies Anti-3-Nitrotyrosine [39B6] (Abcam 61392) and eNOS type III (BD Biosciences 610296) at 1:100 dilution were applied overnight. A no primary antibody control was completed to detect non-specific protein binding. Samples were subsequently incubated with anti-mouse IgG for 1 h (Immpress HRP reagent kit, MP-7452 Vector laboratories). Diaminobenzidine (DAB) (BD Biosciences 550880) was applied as a chromogen before counterstaining with hematoxylin, dehydration, and mounting in Dibutylphthalate Polystyrene Xylene (DPX) [24].

### 2.5. IHC Semiquantification

Images of each vessel were taken at 40× magnification (Leica DFC 450F, Leica Microsystems, Wetzlar, Germany). The endothelium was traced and the degree of staining (brown from DAB) was quantified using the MCID programme (MCID 7.0, Interfocus, Linton, UK). Researchers were blinded to the samples for quantification, using methods previously established [25,26,27,28,29,30]. The proportional intensity (arbitrary unit) of staining was calculated as a ratio of colour intensity to proportional area, normalised to the no primary antibody control. Finally, the immunoreactivity of each protein was calculated based on a fold change from the respective control vessel (the control ring from the normal diet or atherogenic diet groups).

### 2.6. Statistical Analysis

All results were expressed as mean ± standard error of the mean (SEM). Unpaired Student’s t test was used for comparison between the diets. A one-way analysis of variance (ANOVA) was used to analyse the comparison between glucose incubations and *Post-hoc* analysis was completed using Fisher’s least significance difference (LSD) test to identify the differences between groups. Data was analysed in Graphpad prism (version 7.1, Graphpad Software, San Diego, CA, USA). *p* < 0.05 was considered statistically significant, trends were reported when *p* = 0.05–0.099, and >0.099 was considered not significant (n/s). Effect sizes are commonly used to study the clinical relevance of an intervention and show the magnitude of the effect that it is producing [31,32,33]. The Cohen’s d (d) equation was used to examine the magnitude of the effect of the high glucose incubations on blood vessel relaxation and immunohistochemistry results. A large effect is considered when d is >0.8, a medium effect between 0.5 and 0.79, and a small effect between 0.2 and 0.49 [34].

## 3. Results

**Figure 1:**
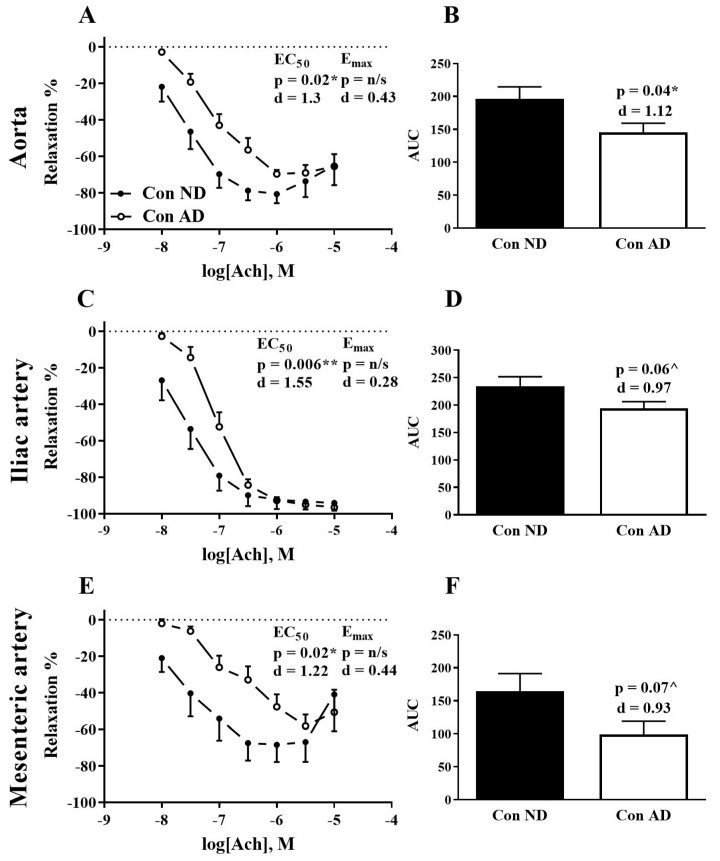
Ach-induced dose response curves in abdominal aorta (**A**), iliac artery (**C**), and mesenteric artery (**E**) incubated ex vivo for 2 h. Comparison between normal diet (closed circles) and atherogenic diet (open circles). Inset: EC_50_ and E_max_ statistical significance (*p*) and effect size (*d*) between diets. AUC in abdominal aorta (**B**), iliac artery (**D**), and mesenteric artery (**F**) presented as arbitrary values; numbers above columns represent the statistical significance (*p*) and effect size (*d*) between diets. *n* = 7–12 per group. All data mean ± SEM. * *p* < 0.05 ND vs. AD, ** *p* < 0.01 ND vs AD, ^ *p* 0.05–0.09 ND vs. AD. ND: normal diet; AD: atherogenic diet; Con: normal Krebs, AUC: area under the curve, d: Cohen’s d.

The atherogenic diet significantly reduced the relaxation of the abdominal aorta as measured by AUC (25%, *p* < 0.05) and EC_50_ (*p* < 0.05) compared to the normal diet (Figure 1A,B). In the iliac artery, the atherogenic diet reduced EC_50_ (*p* < 0.05) and there was a strong trend for a reduction in AUC (17%, *p* = 0.06) compared to the normal diet (Figure 1C,D). Similarly, in the mesenteric artery, the atherogenic diet shifted EC_50_ to the right (*p* < 0.05) and there was a strong trend for a reduction in AUC (40%, *p* = 0.07) (Figure 1E,F).

**Figure 2:**
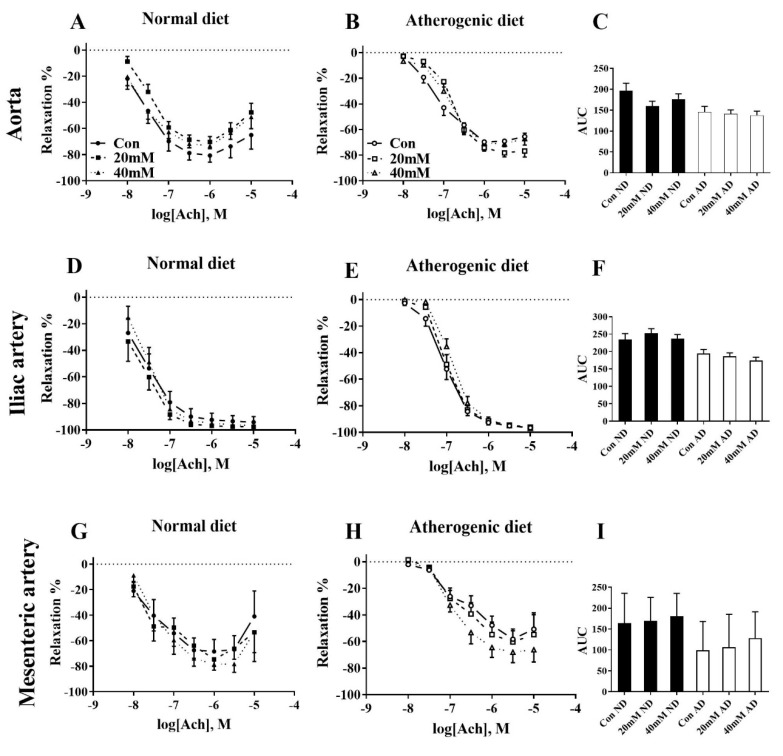
Ach-induced endothelium-dependent dose response curves in abdominal aorta (**A**,**B**), iliac artery (**D**,**E**), and mesenteric artery (**G**,**H**) incubated ex vivo for 2 h in respective solution. Comparison between Con (circles + line), 20 mM (squares + dashes), and 40 mM (triangles + dots). AUC (**C**,**F**,**I**) presented as arbitrary values. *n* = 6–12 per group. All data mean ± SEM. Con: normal Krebs; 20 mM: 20 mM glucose Krebs; 40 mM: 40 mM glucose Krebs; ND: normal diet; AD: atherogenic diet; AUC: area under the curve; Ach: acetylcholine.

For the rabbits who were fed a normal diet, incubation of the aorta in 20 mM glucose produced a strong trend towards a reduction in AUC (18%, *p* = 0.08) and E_max_ was reduced by 10%, but this was not significant (*p* > 0.1) (Figure 2A,C, Table 1). Incubation of the aorta in 20 mM glucose for the atherogenic diet fed rabbits caused a shift to the right of the dose response curve reducing EC_50_ (*p* < 0.05) (Figure 2B and Table 1). No dysfunction was caused in the iliac artery following the normal diet, irrespective of glucose incubation (Figure 2D,F). Whereas, relaxation of the iliac artery from the atherogenic diet fed animals altered EC_50_ in the 40 mM (*p* < 0.05) incubated group (Figure 2E and Supplementary Table S1). Endothelial dependent relaxation of the mesenteric artery was not negatively affected by the high glucose incubations following either the normal or atherogenic diet (Figure 2G–I and Supplementary Table S1).

**Figure 3:**
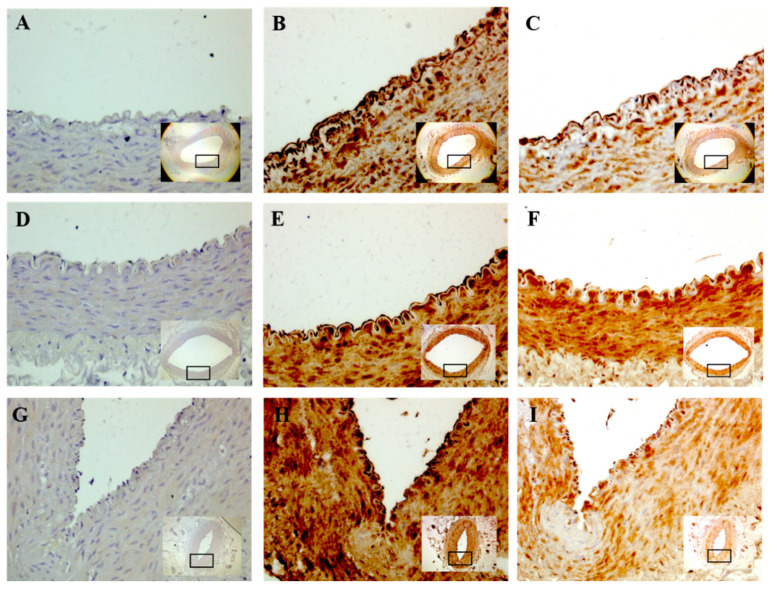
Representative images of immunohistochemistry stained blood vessels; abdominal aorta (**A**–**C**), iliac (**D**–**F**), and mesentery (**G**–**I**) from normal diet fed rabbits. No primary antibody control (**A**,**D**,**G**), nitrotyrosine (NT) (**B**,**E**,**H**), and endothelial nitric oxide synthase (eNOS) (**C**,**F**,**I**) taken at 40× magnification. Inset—image of whole vessel taken at 4× magnification (abdominal aorta) or 10× magnification (iliac and mesentery).

**Figure 4:**
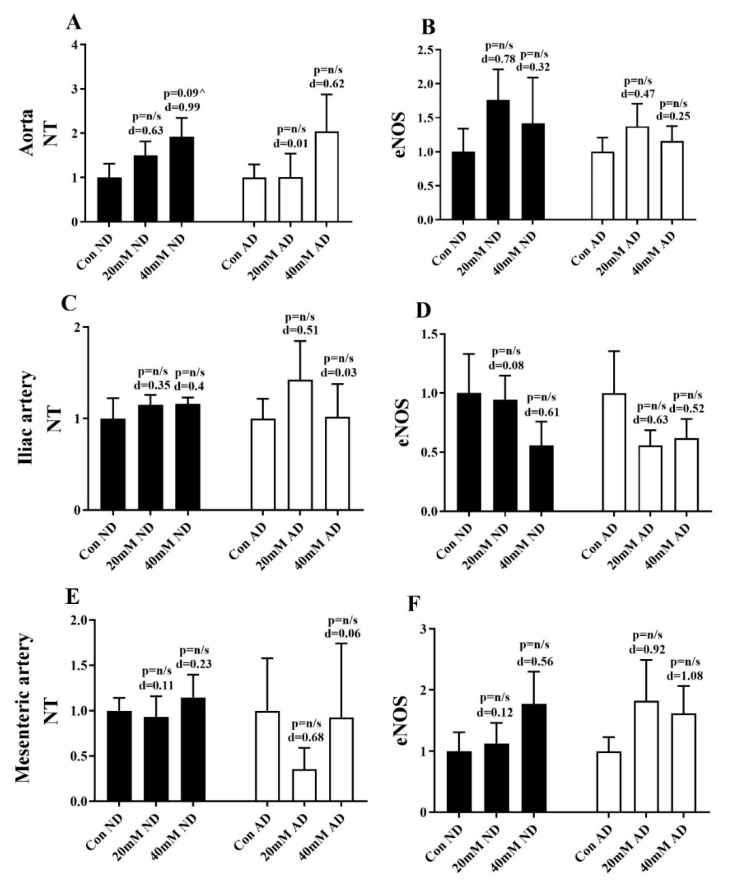
Immunoreactivity of NT and eNOS in abdominal aorta (**A**,**B**), iliac artery (**C**,**D**), and mesenteric artery (**E**,**F**). Immunoreactivity is calculated based on the intensity of the staining present on the endothelium, which is an arbitrary unit and expressed as fold change from the respective control. Numbers above columns represent the statistical significance (*p*) and effect size (*d*) in comparison to the control group for each diet. ^ *p* 0.05–0.99 vs. control. Con: normal Krebs; 20 mM: 20 mM glucose Krebs; 40 mM: 40 mM glucose Krebs; ND: normal diet; AD: atherogenic diet; NT: nitrotyrosine; eNOS: endothelial nitric oxide synthase.

Representative images of IHC stained vessels are presented in Figure 3. The incubation of blood vessels in 20 mM and 40 mM glucose for 2 h did not significantly affect the immunoreactivity of eNOS and NT in any group. NT was increased in the 40 mM glucose normal diet group by 0.9 fold compared to the control, which had a trend towards significance (*p* = 0.9) and a large effect (*d* = 0.99) (Figure 4A). A medium to large effect (*d*) was present in a number of groups, but this was not associated with statistical significance (Figure 4A–F).

## 4. Discussion

We report for the first time that high glucose-induced endothelial dysfunction is blood vessel specific. The abdominal aorta is the most susceptible to high glucose induced dysfunction, with the iliac artery affected to a lesser degree, and the mesenteric artery exhibited no signs of dysfunction.

High fat diets are commonly used to study the development of endothelial dysfunction and atherosclerosis in animals. The 4-week atherogenic diet used in this study has previously been shown to exhibit endothelial dysfunction in abdominal aorta of rabbits [21]. We confirm the findings of atherogenic diet induced endothelial dysfunction in the aorta and demonstrate endothelial dysfunction in the peripheral iliac and mesenteric arteries. Altogether, this suggests that the atherogenic diet functions systemically to cause dysfunction.

Hyperglycaemia is a major clinical risk factor for the development of endothelial dysfunction, atherosclerosis, and CAD. This is the first study to examine the effect of high glucose incubations on endothelial function of blood vessels in various locations. We demonstrate that the abdominal aorta is the artery that is most prone to developing endothelial dysfunction following both the normal and atherogenic diet. This confirms findings from several previous studies, which reported endothelial dysfunction in rat and rabbit aorta following acute high glucose incubations [13,16,35]. The iliac artery exhibited minor high glucose-induced dysfunction following the atherogenic diet, but not following the normal diet. As such, the iliac artery appears to be more susceptible to developing high glucose-induced dysfunction in a disease state and not in a healthy environment. Alternatively, the mesenteric artery did not develop any signs of endothelial dysfunction. Susceptibility to atherosclerosis can depend on haemodynamic factors such as shear stress and oscillating flow, which can vary between vascular sites depending on the location of arterial branches or bifurcations [36]. The exposure of the endothelium to low shear stress is one of the most important factors in atherosclerosis development and is an important consideration when examining endothelial dysfunction in vivo [37]. Furthermore, endothelial dysfunction is not a systemic condition and some blood vessels can often resist the development of dysfunction more than others [38]. For example, vascular beds such as the internal mammary artery and other conduit arteries have increased NO production, decreased vasoconstriction, and have higher shear stress than other vessels [38,39]. Overall, there is variance in the effect of the high glucose incubations on endothelial function in different blood vessels, which may be explained, at least in part, by variations in the structure, physiological effects, and disease susceptibility of each vessel.

In this study, the development of endothelial dysfunction to high glucose incubation was not dose-dependent. The 20 mM glucose incubation caused the largest reduction in endothelium dependent vasodilation in the aorta from both the normal diet fed and atherogenic diet fed rabbits. This finding is in contrast with a previous study, which reported that incubation of rabbit aorta in 44 mM glucose aggravated dysfunction compared to the 20 mM incubation [13]. Similarly, the relaxation of the third order branches of the mesenteric artery from female Wistar rats following incubations in 20 mM and 45 mM glucose solution for 2 h elicited a dose-dependent reduction in endothelial-dependent vasodilation [19]. The conflicting results in this study possibly occurred as a result of species or methodological differences. Taken together, this study demonstrates endothelial dysfunction in the aorta following 2 h high glucose incubations in the normal and atherogenic diets. The dysfunction caused by the 2 h 20 mM glucose incubation provides a model for studying high glucose-induced blood vessel dysfunction that mimics an acute post-prandial response.

In a normal physiological environment, eNOS synthesises NO, which has a number of anti-atherogenic functions including vasodilation [40]. An acute state of hyperglycaemia can reduce eNOS expression and subsequently NO bioavailability, resulting in endothelial dysfunction [41]. Hyperglycaemia also promotes electron transport system overproduction of superoxide anion and via signalling pathways, produces peroxynitrite, a potent ROS [42]. Mechanistically, NT is used as a marker of peroxynitrite production, indicating the presence of nitrative stress [42]. Although not significant, the increase in NT observed in the aorta following high glucose incubations suggests the presence of nitrative stress in the current study—an effect that has previously been reported in rabbit aorta in a disease state [43]. Several recent studies, in both human and animal models, have identified that increased fasting glucose levels as a result of a high fat diet cause reductions in eNOS and plasma nitrate [44,45]. Overall, the evidence suggests that an increase in oxidative/nitrative stress and a reduction in eNOS are characteristic of hyperglycaemia-induced dysfunction. In this study, we did not find any significant alterations in NT or eNOS, but moderate to large changes in the effect size suggests that future research should examine this in more detail.

A potential limitation of the current study is that superoxide anion or other ROS forms were not directly measured to determine the exact mechanistic effect of the high glucose incubations. Furthermore, NT alone may not provide the most accurate representation of hyperglycaemia induced oxidative stress as it may be influenced by other factors including the atherogenic diet [46]. We examined total eNOS expression in combination with NT as an indirect measure of superoxide overproduction and peroxynitrite induced oxidative stress.

In conclusion, the effect of acute high glucose incubations on blood vessel function is blood vessel specific and in some cases, is aggravated by an atherogenic diet. The abdominal aorta may be the optimal artery to study potential therapeutic treatments of hyperglycaemia-induced endothlial dysfunction and CAD in rabbit models.

## Figures and Tables

**Table 1:** Log EC_50_, E_max_ and AUC results from ND and AD fed rabbits incubated ex vivo for 2 h in control, 20 mM, or 40 mM glucose solution.

Abdominal Aorta	*n*	Log EC50 ± SEM	*p* vs. Con	*p* vs. Con	Emax ± SEM	*p* vs. Con	*d* vs. Con	AUC ± SEM	*p* vs. Con	*d* vs. Con
ND Con	7	−7.59 ± 0.12			−81 ±13			196 ± 18		
ND 20 mM	7	−7.43 ± 0.09	n/s	0.54	−70 ± 11	n/s	0.32	160 ± 11	**0.08 ^**	0.92
ND 40 mM	7	−7.65 ± 0.1	n/s	0.18	−73 ± 2	n/s	0.29	177 ± 12	n/s	0.48
AD Con	10	−7.10 ± 0.13			−70 ± 2			146 ± 13		
AD 20 mM	11	−6.81 ± 0.06	0.03 *	0.88	−78 ± 3	0.04 *	0.97	141 ± 9	n/s	0.13
AD 40 mM	11	−6.93 ± 0.07	n/s	0.52	−71 ± 3	n/s	0.18	138 ± 10	n/s	0.21

ND: normal diet; AD: atherogenic diet; Con: normal Krebs; 20 mM: 20 mM glucose Krebs; 40 mM: 40 mM glucose Krebs; 2 h: 2 h incubation; AUC: area under the curve; *d*: Cohen’s d; *n* = number of rabbits. Statistical significance (*p*) and effect size (Cohen’s d) in comparison to the control group for each diet. * *p* < 0.05 vs. control, ^ *p* 0.05–0.99 vs. control.

## Supplementary Materials

The following are available online at https://www.mdpi.com/2072-6643/12/7/2108/s1. Table S1: Log EC50, Emax and AUC results from ND and AD fed rabbits incubated ex vivo for 2 hr in control, 20 mM or 40 mM glucose solution.
