# Supplementary material for: The Effect of an Atherogenic Diet and Acute Hyperglycaemia on Endothelial Function in Rabbits Is Artery Specific
Source: Nutrients. 2020 Jul 16;12(7):2108. doi: 10.3390/nu12072108 (PMC7400854; doi:10.3390/nu12072108)
Supplement: Supplementary file 1 [file nutrients-12-02108-s001.pdf]

Supplementary Table 1. Log EC_{50}, E_{max} and AUC results from ND and AD fed rabbits incubated ex vivo for 2hr in control, 20mM or 40mM glucose solution.

**Iliac artery**		**Log EC_{50} ± SEM**	**p vs con**	**d vs Con**	**E_{max} ± SEM**	**p vs con**	**d vs Con**	**AUC ± SEM**	**p vs con**	**d vs con**
ND Con	7	-7.52 ± 0.12			-94 ± 4			235 ± 17		
ND 20mM	7	-7.64 ± 0.11	n/s	0.41	-98 ± 1	n/s	0.43	252 ± 14	n/s	0.42
ND 40mM	6	-7.55 ± 0.13	n/s	0.1	-97 ± 2	n/s	0.31	237 ± 12	n/s	0.05
AD Con	10	-7.07 ± 0.08			-97 ± 1			194 ± 12		
AD 20mM	11	-7.00 ± 0.06	n/s	0.32	-96 ± 1	n/s	0.11	186 ± 10	n/s	0.23
AD 40mM	10	-6.85 ± 0.06	**0.03***	0.98	-97 ± 1	n/s	0.09	175 ± 9	n/s	0.59
**Mesenteric artery**		**Log EC_{50} ± SEM**	**p vs con**	**d vs Con**	**E_{max} ± SEM**	**p vs con**	**d vs Con**	**AUC ± SEM**	**p vs con**	**d vs con**
ND Con	7	-7.44 ± 0.19			-68 ± 9			164 ± 27		
ND 20mM	6	-7.46 ± 0.23	n/s	0.04	-75 ± 6	n/s	0.3	170 ± 23	n/s	0.08
ND 40mM	6	-7.35 ± 0.18	n/s	0.2	-79 ± 5	n/s	0.52	181 ± 22	n/s	0.26
AD Con	12	-6.74 ± 0.18			-58 ± 6			99 ± 20		
AD 20mM	11	-6.50 ± 0.18	n/s	0.4	-60 ± 10	n/s	0.08	107 ± 24	n/s	0.11
AD 40mM	11	-6.81 ± 0.12	n/s	0.12	-68 ± 8	n/s	0.41	128 ± 19	n/s	0.45

ND: normal diet; AD: atherogenic diet; Con: normal Krebs; 20mM: 20mM glucose Krebs; 40mM: 40mM glucose Krebs; 2hr: 2 h incubation; AUC: area under the curve; d: Cohen's d; n = number of rabbits.

Statistical significance (p) and effect size (Cohen's d) in comparison to the control group for each diet. *p < 0.05 vs control.
